# The NIMO Scandinavian Study: A Prospective Observational Study of Iron Isomaltoside Treatment in Patients with Iron Deficiency

**DOI:** 10.1155/2017/4585164

**Published:** 2017-10-22

**Authors:** Svein Oskar Frigstad, Anne Haaber, Antal Bajor, Jan Fallingborg, Per Hammarlund, Ole K. Bonderup, Håkan Blom, Terje Rannem, Per M. Hellström

**Affiliations:** ^1^Department of Medicine, Bærum Hospital, Vestre Viken Hospital Trust, Drammen, Norway; ^2^Institute of Clinical Medicine, University of Oslo, Oslo, Norway; ^3^Department of Gastroenterology, Gentofte Hospital, Hellerup, Denmark; ^4^Department of Internal Medicine, Södra Älvsborg Hospital, Borås, Sweden; ^5^Department of Gastroenterology and Hepatology, Aalborg University Hospital, Aalborg, Denmark; ^6^Department of Gastroenterology, Ängelholm Hospital, Ängelholm, Sweden; ^7^Department of Gastroenterology, Silkeborg Regional Hospital, Silkeborg, Denmark; ^8^Department of Medicine, Sunderby Hospital, Luleå, Sweden; ^9^Department of Gastroenterology, Nordsjælland Hospital, Frederikssund, Denmark; ^10^Department of Medical Sciences, Uppsala University, Uppsala, Sweden

## Abstract

**Background:**

Intravenous iron allows for efficient and well-tolerated treatment in iron deficiency and is routinely used in diseases of the gastrointestinal tract.

**Objective:**

The aims of this study were to determine the probability of relapse of iron deficiency over time and to investigate treatment routine, effectiveness, and safety of iron isomaltoside.

**Methods:**

A total of 282 patients treated with iron isomaltoside were observed for two treatments or a minimum of one year.

**Results:**

Out of 282 patients, 82 had Crohn's disease and 67 had ulcerative colitis. Another 133 patients had chronic blood loss, malabsorption, or malignancy. Patients who received an iron isomaltoside dose above 1000 mg had a 65% lower probability of needing retreatment compared with those given 1000 mg. A clinically significant treatment response was shown, but in 71/191 (37%) of patients, anaemia was not corrected. The mean dose given was 1100 mg, lower than the calculated total iron need of 1481 mg. Adverse drug reactions were reported in 4% of patients.

**Conclusion:**

Iron isomaltoside is effective with a good safety profile, and high doses reduce the need for retreatment over time. Several patients were anaemic after treatment, indicating that doses were inadequate for full iron correction. This trial is registered with NCT01900197.

## 1. Introduction

Intravenous (IV) iron allows for efficient and well-tolerated treatment in iron deficiency and is routinely used in diseases of the gastrointestinal tract. In inflammatory bowel disease (IBD), iron deficiency anaemia is the most common systemic complication and has been reported in 20–42% of patients [1–7], while iron deficiency has been reported in 35–90% of patients [1, 2, 6, 8–10]. Anaemia has been shown to negatively affect health-related quality of life and contribute to chronic fatigue in IBD patients [9, 11, 12].

According to current guidelines, iron supplementation should be administered in all cases of IBD with iron deficiency anaemia and to prevent recurrence of anaemia after prior iron supplementation [13]. In iron deficiency without anaemia, an individualised approach is recommended [13, 14]. The use of oral iron supplementation has limitations in clinical practice, including reduced efficiency, poor tolerance, and low adherence, and thus, IV iron is widely recommended [13, 15–17]. IBD patients with iron deficiency usually need high doses of iron, and full iron correction is in such cases only possible with IV iron treatment [13, 18]. Iron deficiency often recurs in IBD patients after iron replacement therapy [13, 18–20]. Additionally, many IBD patients do not receive adequate treatment for iron deficiency, and increased awareness and optimised treatment strategies are needed [3, 6, 21].

Iron isomaltoside 10% (Monofer®, Pharmacosmos A/S, Denmark) is a high-dose IV iron for fast infusion. The strongly bound formulation of iron and the carbohydrate isomaltoside in a matrix structure enables a controlled slow release of iron with small risk of iron toxicity. This allows for administration of high doses of iron in one visit. More than 3000 patients have been treated with iron isomaltoside in clinical studies until now, and more than 5.3 million doses have been used in clinical practice (data on file, Pharmacosmos A/S, Denmark).

The aims of this study were to determine the probability of relapse of iron deficiency over time, defined as retreatment related to the dose given, and further to investigate treatment routine, effectiveness, and safety of iron isomaltoside in clinical practice.

## 2. Materials and Methods

### 2.1. Study Design and Population

Participants were recruited from a total of 10 sites in Denmark, Norway, and Sweden in a prospective, observational study conducted from August 2013 to November 2015. Inclusion criteria were age > 17 years and diagnosed with iron deficiency anaemia following local clinical guidelines. The patients were treated with iron isomaltoside according to the product label and clinical routine. The patients were followed prospectively for two treatments of iron isomaltoside given or for a minimum of 12 months after the first treatment. For patients not receiving two treatments, the observation time lasted until completion of the study in order to capture a second treatment. Due to the observational design of the study, all procedures were done according to local clinical practice and decisions by the participating investigators.

### 2.2. Data Collection

Data for iron isomaltoside treatment were collected prospectively for up to two treatments per patient. The dose administered in the study and the calculated iron need [13, 22] were recorded. Use of concomitant medication and pre- and posttreatment blood tests was also recorded. Data were collected from medical records. Treatment response was defined as normalisation of haemoglobin (Hb) or increase in Hb ≥ 2 g/dL [23, 24]. The occurrence of adverse drug reactions (ADRs) was registered and reported to the sponsor's pharmacovigilance department and according to national reporting systems. The collected data were systematically entered into an electronic case report form (eClinicalOS, Merge Healthcare, NC, USA; licensed by BioStata ApS, Denmark).

### 2.3. Statistical Methods

**Figure 1:**
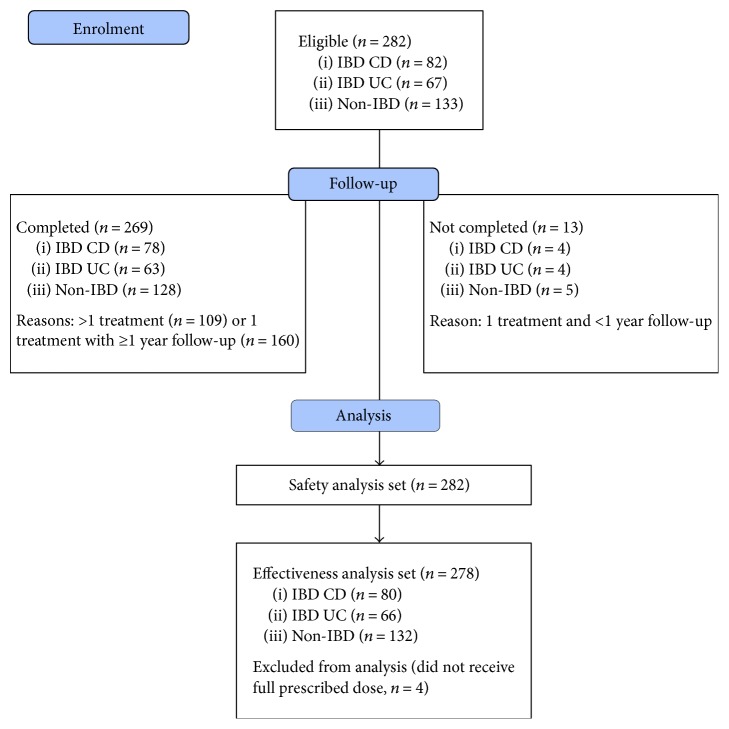
Patient flow diagram (based on CONSORT 2010). CD: Crohn's disease; IBD: inflammatory bowel disease; UC: ulcerative colitis.

The safety analysis set population (*n* = 282) included all patients who were enrolled in the study and received the study drug, and the effectiveness analysis set population (*n* = 278) included all patients who received a full prescribed dose. The patient flow diagram is shown in Figure 1.

Data are presented with mean and standard deviation or median and range, for continuous variables, and with a number of exposed subjects with percentages for categorical variables. The probability of iron isomaltoside retreatment over time was analysed using a Cox proportional hazards model and was determined from survival plots. Laboratory parameter changes were obtained from an analysis of covariance model. The effect of the iron dose on the Hb response was analysed by logistic regression. The significance cut-off for all analyses was *p* < 0.05. No formal adjustment for multiplicity was performed. Data were analysed using SAS version 9.4 (SAS Institute, USA).

### 2.4. Ethical Considerations

The Regional Ethics Committee in Sweden (EPN Lund 2013/231), the Data Protection Official for Research in Norway (2013/10419), and the Danish Data Protection Agency (2013–41-1543) approved the study. The study was registered at ClinicalTrials.gov (NCT01900197). All study participants gave written, informed consent before inclusion, and the study was performed in accordance with the Declaration of Helsinki and the European Medicines Agency criteria for noninterventional studies [25].

## 3. Results

### 3.1. Patients

A total of 282 patients were included in the study, and of these, 82 (29%) had Crohn's disease (CD) and 67 (24%) had ulcerative colitis (UC). A remaining group of 133 (47%) non-IBD patients was iron deficient due to chronic blood loss, malabsorption, or malignancy. At baseline, the use of concomitant medications for anaemia correction was low (oral iron 13%, blood transfusion 3%, erythropoiesis-stimulating agents 0.4%, and cobalamin or folic acid 28%). Baseline characteristics of the study population are summarised in Table 1. As shown, most patients having available ferritin data at baseline (both IBD and non-IBD patients) were iron deficient with a ferritin cut-off < 100 *μ*g/L for iron deficiency. Not enough data for CRP were collected to be able to assess inflammation status.

### 3.2. Probability of Retreatment and Treatment Routine

Of the 278 patients receiving a full prescribed dose of iron isomaltoside, 53 (19%) were given >1000 mg, 186 (67%) were given 1000 mg, and 39 (14%) were given <1000 mg at the first treatment. In 264/278 (95%) of patients, the full prescribed dose was administered in one single visit for the first iron isomaltoside treatment.

**Figure 2:**
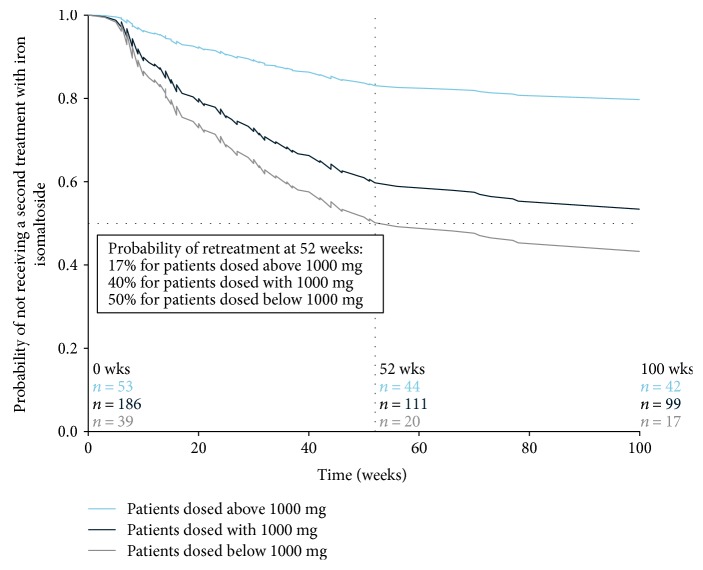
Probability of retreatment related to iron isomaltoside dose. The survival plot was obtained by using the Cox proportional hazards model with dose and diagnosis as factors and baseline haemoglobin as covariate. The probability (percent) of retreatment with iron isomaltoside at 52 weeks after the first treatment for each dose group was calculated from the survival curve. The number of patients at weeks 0, 52, and 100 for each dose group includes all patients.

The majority of patients, 170/278 (61%), received only one treatment of iron isomaltoside during a median observation time of 19 (1–27) months. In 108/278 (39%) of patients who were retreated, those who received a dose > 1000 mg at the first treatment had a 65% lower probability (hazard ratio: 0.351; 95% confidence interval (CI): 0.19, 0.66) of needing retreatment compared to those given 1000 mg. For patients who received a dose < 1000 mg at the first treatment, there was 36% higher probability (hazard ratio: 1.358; 95% CI: 0.81, 2.27) of needing retreatment compared to patients given 1000 mg. The hazard ratios were corrected for dose, diagnosis, and baseline Hb. The probability of retreatment at 52 weeks split by dose is shown in Figure 2.

In addition to the administered dose, baseline Hb was an independent predictor of the probability for retreatment, where the need for retreatment decreased by 18% (hazard ratio: 0.825; 95% CI: 0.74, 0.90) for each 1 g/dL unit higher baseline Hb. When comparing patients with CD and UC to non-IBD patients, there were no significant differences in the need for retreatment.

### 3.3. Effectiveness

**Figure 3:**
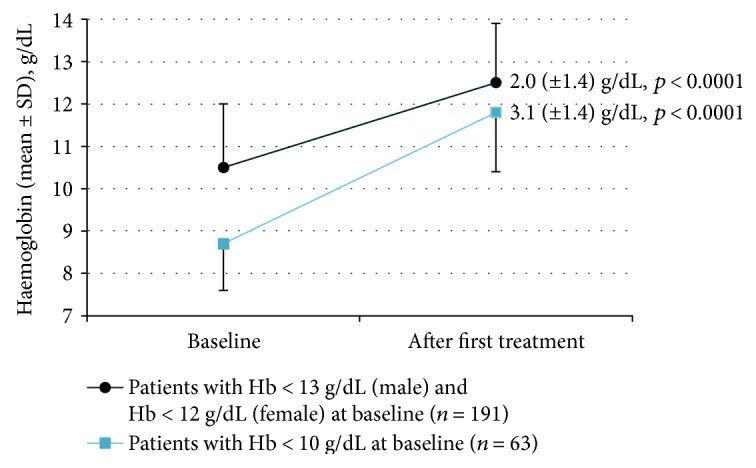
Change in haemoglobin (Hb) after the first iron isomaltoside treatment in anaemic patients with available pre- and posttreatment Hb measures. The time of evaluation corresponded to the time between the last administration of first iron isomaltoside treatment and blood test follow-up and occurred during a mean (SD) time of 7.1 (6.0) weeks for anaemic patients at baseline and during a mean (SD) time of 6.1 (4.7) weeks for patients with Hb < 10 g/dL at baseline. The *p* value calculations were obtained by analysis of covariance test with diagnosis and dose as factors and pretreatment laboratory parameter concentration as covariate.

Effectiveness was assessed around 7 weeks after the first iron isomaltoside treatment (see Table 2 and Figure 3). Treatment response following the first treatment was achieved in 143/191 (75%) of the patients who were anaemic at baseline. The response rate after the first treatment increased significantly with an increasing iron isomaltoside dose (8/14 [57%] for doses < 1000 mg, 93/128 [73%] for doses of 1000 mg, and 42/49 [86%] for doses > 1000 mg) even when correcting for baseline Hb (*p* < 0.05). Of patients with anaemia at baseline, 71/191 (37%) were still anaemic following the first treatment.

### 3.4. Dosing in Clinical Practice and Calculated Iron Need

The calculated iron need using the Ganzoni formula and simplified dosing compared to the actual dose given in the study are shown in Table 3.

### 3.5. Safety

Twelve ADRs were reported in 10/282 (4%) of the patients given a total of 408 high-dose iron isomaltoside administrations. ADRs were seen in seven IBD patients and three non-IBD patients. Infusion reactions were reported in 6/282 (2%) of patients; the rest were delayed hypersensitivity reactions. One of the patients was admitted to the hospital for the treatment of a serious acute infusion reaction with generalised erythema, dyspnoea, general body pain mainly localised to the abdomen, and hypotension. We found no association between dose given and ADRs reported. All patients had uneventful recoveries.

## 4. Discussion

The main result of this study was that the probability of retreatment with IV iron could be reduced with dosing above 1000 mg. Most patients were given doses of 1000 mg or higher, and the majority received their full prescribed dose in one single visit and were treated only once during the observation period. The administered dose of iron isomaltoside and baseline Hb were independent predictors for retreatment. The response rate increased with higher dose; however, several patients remained anaemic after treatment indicating that patients receive inadequate iron dosing in routine clinical practice.

To our knowledge, no other studies have shown that higher doses of IV iron reduce the need for retreatment over time. In a Swedish observational study, it was demonstrated that the effect of IV iron treatment in IBD patients was mostly sustained for a year with up to 29% of patients being retreated within this period. In this study, however, association to an administered dose was not evaluated [26]. In an Austrian retrospective study following IBD patients treated with iron sucrose, the dose given had no influence on the need for retreatment over time [19].

In line with other studies, we found that the response rate correlated to the iron dose given [19, 20, 27]. There are few observational studies of IV iron in clinical practice in IBD [26, 28–30]. None of these have evaluated the dose given compared to the dose needed for full iron correction. In our study, we found that lower doses than the calculated iron need as recommended by international guidelines were given in clinical practice [13].

Treatment with IV iron in the current study showed a good safety profile with a frequency of hypersensitivity reactions similar to that reported in other studies even if high doses were given to the majority of our patients [15, 18–20, 26–29, 31, 32].

The limitation of our study is the observational design, but one of the aims of the study was to observe treatment routines in clinical practice to provide guidance for optimised treatment strategies for IV iron treatment. Hence, no instructions were given on dosing, time to effectiveness assessment, and retreatment. We did not collect data on clinical disease activity or anti-inflammatory treatment, which can influence recurrence of anaemia and the need for iron retreatment in patients with IBD.

Taken together, iron isomaltoside was effective with a good safety profile in both IBD and non-IBD patients with iron deficiency. A high dose, especially over 1000 mg, reduced the need for retreatment. The administration of higher doses, as recommended in current guidelines, seems required for the full iron correction and prevention of iron deficiency anaemia.

## 5. Conclusions

In this study, we show that iron isomaltoside allows for efficient and well-tolerated treatment in iron deficiency and furthermore that high doses reduce the need for retreatment over time. Several patients were anaemic after treatment, indicating that doses routinely given were inadequate for full iron correction. Infusion reactions were reported in 2% of patients, similar to what has been reported in clinical trials.

## Figures and Tables

**Table 1:** Demographic and baseline characteristics.

	IBD CD (*n* = 82)	IBD UC (*n* = 67)	Non-IBD (*n* = 133)	Total (*n* = 282)
*n* (%)				
Gender				
Female	46 (56.1)	34 (50.7)	93 (69.9)	173 (61.3)
Male	36 (43.9)	33 (49.3)	40 (30.1)	109 (38.7)
Anaemic^a^ patients	(*n* = 82)	(*n* = 67)	(*n* = 132)	(*n* = 281)
	56^b^ (68.3)	44^b^ (65.7)	98 (74.2)	198 (70.5)
Patients with Hb < 10 g/dL	(*n* = 82)	(*n* = 67)	(*n* = 132)	(*n* = 281)
	14 (17.1)	11 (16.4)	41 (31.1)	66 (23.5)
*Mean (SD)*				
Age, years	(*n* = 82)	(*n* = 67)	(*n* = 133)	(*n* = 282)
	42.2 (16.2)	41.1 (15.9)	54.3 (18.6)	47.6 (18.4)
Weight, kg	(*n* = 78)	(*n* = 64)	(*n* = 132)	(*n* = 274)
	73.1 (17.3)	76.4 (15.4)	74.6 (16.4)	74.6 (16.4)
Hb, g/dL	(*n* = 82)	(*n* = 67)	(*n* = 132)	(*n* = 281)
	11.6 (1.7)	11.5 (1.7)	10.9 (1.9)	11.3 (1.8)
TSAT, %	(*n* = 55)	(*n* = 41)	(*n* = 59)	(*n* = 155)
	9.9 (7.1)	9.4 (6.5)	10.3 (7.3)	9.9 (7.0)
Ferritin, *μ*g/L	(*n* = 70)	(*n* = 63)	(*n* = 117)	(*n* = 250)
	34.3 (59.8)	28.3 (59.1)	23.9 (84.9)	27.9 (72.4)
Median (IQR)^c^	11.0 (6.2–27)	11.0 (7.0–21)	9.0 (5.6–16)	10.0 (6.0–20)

^a^Hb < 13 g/dL (men) and Hb < 12 g/dL (women). ^b^Mean (SD) Hb in IBD patients 10.8 (1.4) g/dL and mean (SD) weight 75.4 (17.4) kg. ^c^Median is presented for data not normally distributed. CD: Crohn's disease; Hb: haemoglobin; IBD: inflammatory bowel disease; IQR: interquartile range; SD: standard deviation; TSAT: transferrin saturation; UC: ulcerative colitis.

**Table 2:** Change in iron and blood cell parameters after the first iron isomaltoside treatment.

	Pretreatment	Posttreatment	Concentration change	*p* value^a^	Time of evaluation^b^
*Mean (SD)*					
Hb, g/dL (*n* = 191)^c^	10.5 (1.5)	12.5 (1.4)	2.0 (1.4)	<0.0001	7.1 (6.0)
TSAT, % (*n* = 108)^d^	9.6 (6.5)	21.0 (9.8)	11.4 (10.0)	<0.0001	7.6 (5.0)
Ferritin, *μ*g/L	25.0 (68.0)	146.3 (173.1)	121.3 (162.6)	<0.0001	7.4 (6.5)
Median (IQR)^e^ (*n* = 222)^d^	10.0 (6.0–20)	95.0 (49–181)	71.0 (35–157)		

^a^ *p* values for concentration changes obtained by analysis of covariance test with diagnosis and dose as factors and pretreatment laboratory parameter concentration as covariate. ^b^Time (weeks) between the last administration of first iron isomaltoside treatment and blood test follow-up. ^c^Anaemic patients (Hb < 13 g/dL for men and Hb < 12 g/dL for women at baseline) with available pre- and posttreatment Hb measures in the total population. ^d^Patients with available pre- and posttreatment laboratory measures in the total population. ^e^Median is presented for data not normally distributed. Hb: haemoglobin; IQR: interquartile range; SD: standard deviation; TSAT: transferrin saturation.

**Table 3:** Study dose versus calculated iron need for anaemic patients.

Anaemic patients^a^	IBD CD (*n* = 50)	IBD UC (*n* = 40)	Non-IBD (*n* = 95)	Total (*n* = 185)
*Mean (SD)*				
Study dose^b^, mg	1058 (338.1)	1115 (265.6)	1115 (305.6)	1100 (306.2)
Iron need calculated from simplified dosing table, mg	1420 (325.1)	1438 (324.0)	1532 (316.3)	1481 (322.9)
Iron need calculated from Ganzoni formula^c^, mg	1268 (279.4)	1261 (316.0)	1380 (284.1)	1324 (294.2)

^a^Anaemia defined as Hb < 13 g/dL for men and Hb < 12 g/dL for women at baseline. Patients having a weight < 50 kg and patients with missing weight data were excluded. ^b^Study dose for first iron isomaltoside treatment. ^c^Ganzoni formula based on a target Hb of 15 g/dL, Hb level before first iron isomaltoside treatment in study, and iron store of 500 mg. CD: Crohn's disease; Hb: haemoglobin; IBD: inflammatory bowel disease; SD: standard deviation; UC: ulcerative colitis.
